# Hypercoagulability Due to COVID-19 Leading to Impending Phlegmasia Cerulea Dolens and Sub-Massive Bilateral Pulmonary Embolism

**DOI:** 10.7759/cureus.17351

**Published:** 2021-08-21

**Authors:** Bruno Moraes, Amir Hashemi, Kevin Mancheno, Manuel ObanDo, Erin Marra

**Affiliations:** 1 Emergency Medicine, Graduate Medical Education, Aventura Hospital and Medical Center, Aventura, USA; 2 Emergency Medicine, Nova Southeastern University, Dr. Kiran C. Patel College of Allopathic Medicine, Aventura, USA

**Keywords:** phlegmasia cerulea dolens, covid-related hypercoagulability, submassive pulmonary embolism, inferior vena cava thrombus, deep vein thrombosis (dvt), interventional radiology guided embolization, ivc filters, d dimer, covid-19, hyper coagulable state

## Abstract

This is a case report of a 47-year-old male with a history of hypertension and pre-diabetes who presented to the emergency department with dyspnea, progressive unilateral leg swelling and pain. The patient tested positive for coronavirus disease 2019 (COVID-19) infection about a week earlier. The patient was found to have an extensive clot burden of his lower extremity veins, both deep and superficial, which extended to his inferior vena cava (IVC). Based on the patient’s clinical exam and ultrasound findings, the patient was diagnosed with impending phlegmasia cerulea dolens. Due to his renal failure, the patient was taken for a ventilation/perfusion (V/Q) scan which found widespread V/Q mismatch highly suggestive of pulmonary embolism. Interventional radiology took the patient for lower extremity venogram, catheter-directed alteplase administration, and IVC filter placement. The patient was admitted to the intensive care unit (ICU) for further management and had a stable recovery.

## Introduction

Deep vein thrombosis (DVT) is a known complication of coronavirus disease 2019 (COVID-19) that has been widely studied. Phlegmasia cerulean dolens is an uncommon but severe complication of DVT. It occurs when an individual has significant venous thrombosis resulting in outflow obstruction with resultant painful lower limb swelling and cyanosis [1]. When left untreated, it may progress to venous gangrene with a high risk for limb loss [1]. It is a condition preceded by phlegmasia alba dolens, where there is significant occlusion of the deep veins of the lower extremity, however, collateral circulation is still present [1]. Present reports of COVID-19 patients with phlegmasia cerulea dolens are rare. However, in none of the reported cases have the patients also had bilateral pulmonary embolism. It has been extensively documented that this novel virus mimics diseases, affects all age groups, and leads to significant morbidity and mortality. Part of the perplexing pathophysiology of COVID-19 is its ability to induce a prothrombotic state but the exact mechanism for causing this hypercoagulability is currently unknown.

Current theories utilize the principles of Virchow’s triad (endothelial injury, hypercoagulability, and blood stasis) which seem to be prevalent in hospitalized patients with COIVD-19 [2]. Patients with pre-existing conditions or comorbidities (i.e., diabetes mellitus, hypertension, obesity, coronary artery disease, chronic liver, and kidney disease) are at much higher risk for extensive venous thrombosis which lead to complications such as venous thromboembolism (VTE) [3]. The presentation of patients with pulmonary embolism in the emergency department and in-patients is an important factor to consider because the signs and symptoms of the embolism can be masked by the many symptoms brought on by the infection. We discuss the management and clinical course of a healthy adult male who tested positive for COVID-19 and was found to have significant clot burden resulting in bilateral sub-massive pulmonary emboli and impending phlegmasia cerulea dolens.

## Case presentation

**Figure 1:**
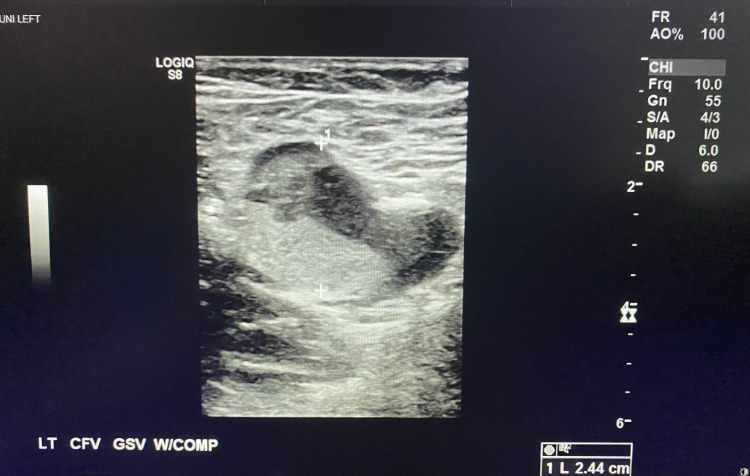
Left Common Femoral and Great Saphenous Veins Non-Compressible with Clot Demonstrated in the Lumen

**Figure 2:**
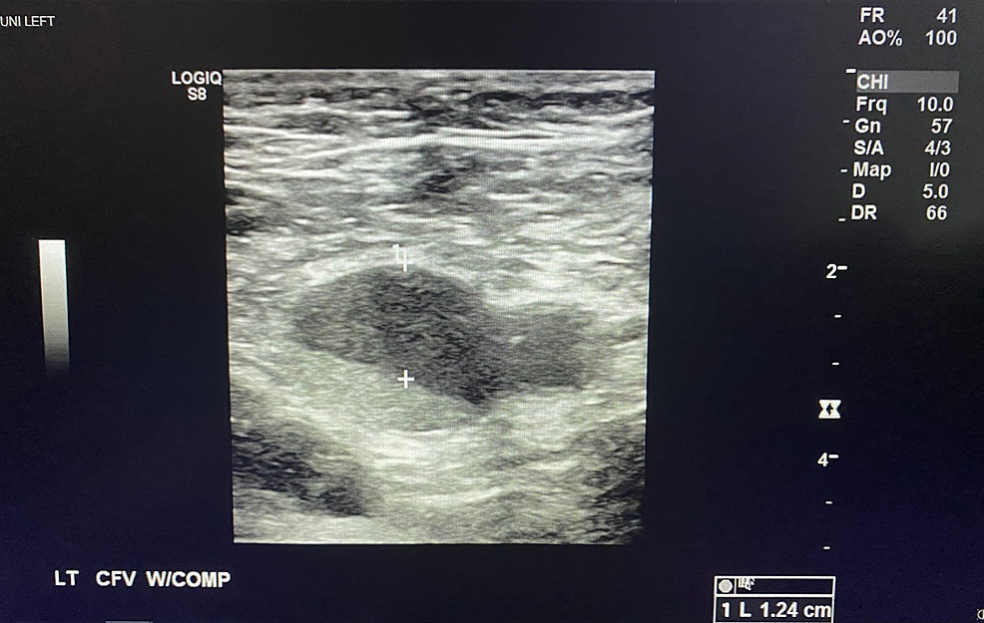
Left Common Femoral Vein Non-Compressible with Clot Demonstrated in the Lumen

**Figure 3:**
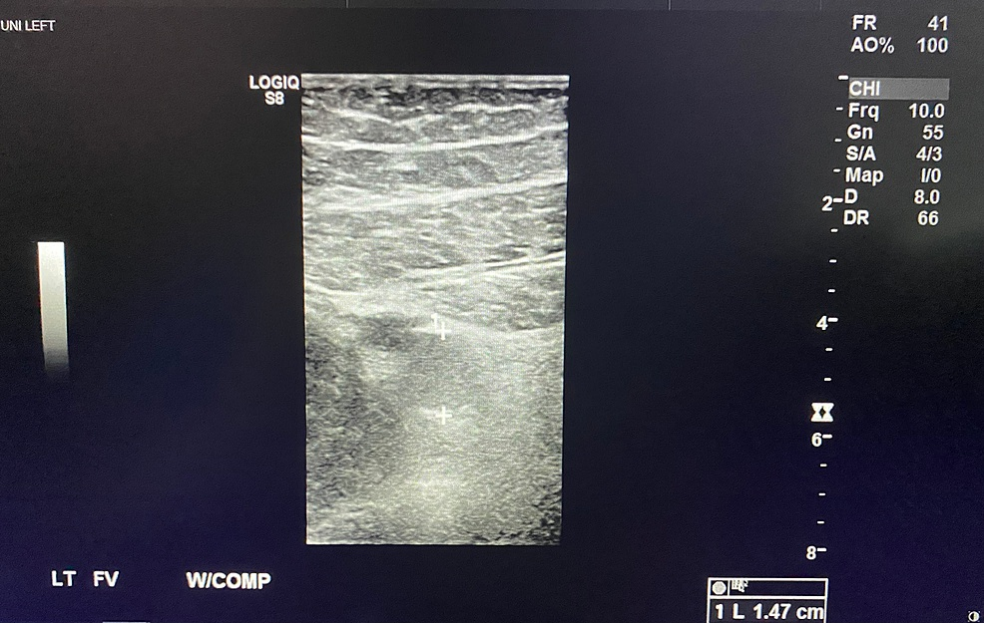
Left Femoral Vein Non-Compressible with Clot Demonstrated in the Lumen

**Figure 4:**
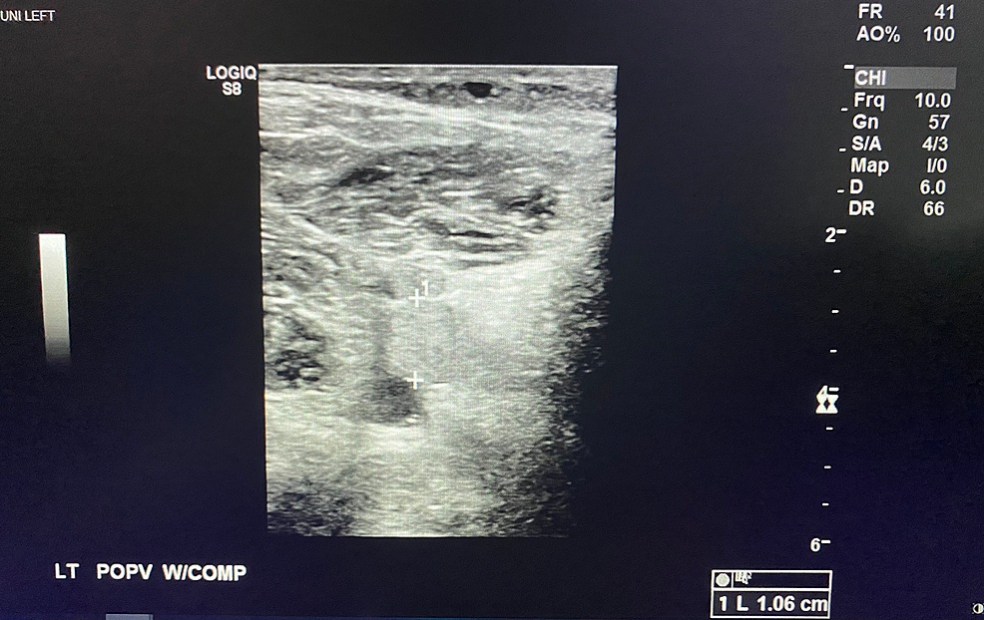
Left Popliteal Vein Non-Compressible with Clot Demonstrated in the Lumen

**Figure 5:**
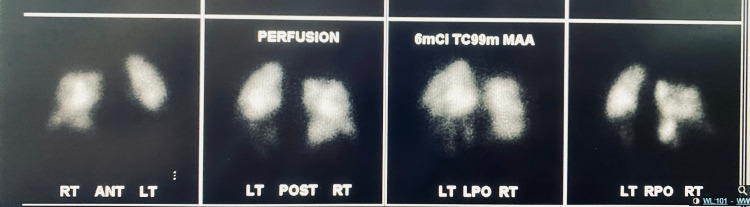
Ventilation-Perfusion Scan Demonstrating Bilateral, Large, Segmental Perfusion Defects

A 47-year-old male with a past medical history of hypertension and pre-diabetes presented to the emergency department with three days of shortness of breath and two days of progressive left lower extremity pain and swelling. The patient reported testing positive for COVID-19 six days prior to presentation. The patient endorsed changes in taste and smell, fatigue, chills, and nausea. There was no prior history of DVT or pulmonary embolism. Physical exam was significant for tachypnea, oxygen saturation of 91% on room air, improved with supplemental oxygen to 100%. The entire left lower extremity was edematous and painful to palpation, with cold digits and non-palpable dorsalis pedis and posterior tibialis pulses, but present with Doppler. A venous duplex ultrasound of the left lower extremity revealed extensive DVT involving the common femoral vein, great saphenous vein, superficial and deep femoral veins, and popliteal vein (Figures 1-4). Given his hypoxia, there were concerns for pulmonary embolism but because the patient had new renal dysfunction, it was decided to proceed with a monitored ventilation/perfusion (V/Q) scan. The V/Q scan revealed multiple, large, segmental perfusion defects in the bilateral lungs representing a high probability for pulmonary embolism (Figure 5). Bedside echocardiogram revealed evidence of acute right heart strain with a dilated right ventricle and paradoxical septal wall motion (Video 1). 

**Video 1:** Parasternal Long Axis View on Transthoracic Echocardiogram Bedside echocardiogram demonstrating right ventricular distension. Evidence of right heart strain secondary to pulmonary embolism.

Interventional radiology was consulted in the emergency department for concerns of phlegmasia cerulea dolens and sub-massive pulmonary embolism. The patient was emergently started on heparin and taken for left lower extremity venography which revealed extensive thrombosis of the femoral system, and thrombosis of the left iliac vein extending into the inferior vena cava (IVC). Interventional radiology took the patient for catheter-directed alteplase administration to the left femoral vein, left pulmonary artery, and main pulmonary artery. They also placed an IVC filter just below the renal veins. The patient stayed in the intensive care unit (ICU) for three days where he received continued alteplase administration, heparin, and supportive care. subsequent echocardiogram showed improvement of right ventricular distention, renal function, and vital signs. The patient was subsequently discharged on a rivaroxaban and appropriate follow up. 

## Discussion

This case demonstrates the hypercoagulability caused by COVID-19. The patient had multi-system involvement with extensive clot burden. His case required emergent intervention with the administration of anti-coagulation, directed thrombolytic medication, and an IVC filter. Literature shows that patients with COVID-19 are at high risk for VTE [4]. Our patient required advanced interventions as noted previously in order to treat his condition. Current retrospective investigation into the efficacy of the Wells’ criteria and Geneva scores reveal that they are not sensitive in patients considered to be critically ill [5]. As a result, these methods do not appear to be reliable in predicting and assessing the need for CT thoracic angiogram studies along with anti-thrombotic therapy in hospitalized COVID-19 patients [5].

At present, the usual finding in COVID-19 patients is an elevated D-dimer with coinciding low platelet levels [6]. Higher D-dimer levels have correlated with higher morbidity and mortality in COVID-19 patients [6]. Treatment with therapeutic dosed heparin was associated with lower 28-day mortality in severe COVID-19 patients who had an elevated D-dimer greater than six fold the upper limit of normal [7]. Our patient's D-dimer value was 16 times the upper limits of normal, so heparin was the appropriate initial choice. In regards to the pathophysiology, the exact mechanism of extensive clot burden remains under investigation. However, current theories suggest that patients with abnormal activity of the complement cascade along with decreased organ function seem to contribute to the hypercoagulable state in COVID-19 [8]. Future research is necessary to create a risk stratification scoring system that will aid in the management and treatment of coagulopathic COVID-19 patients.

## Conclusions

The severe clot burden in this 47-year-old man without risk factors for hypercoagulability was likely secondary to coagulopathy in the setting of COVID-19 infection. Our patient had impending phlegmasia cerulae dolens with sub-massive bilateral pulmonary embolisms which required significant intervention such as direct thrombolysis and anticoagulation. This case highlights the extent of VTE and hypercoagulability seen in a patient dealing with the COVID-19 viral infection.
